# To Fish or Not to Fish: Factors at Multiple Scales Affecting Artisanal Fishers' Readiness to Exit a Declining Fishery

**DOI:** 10.1371/journal.pone.0031460

**Published:** 2012-02-10

**Authors:** Tim M. Daw, Joshua E. Cinner, Timothy R. McClanahan, Katrina Brown, Selina M. Stead, Nicholas A. J. Graham, Joseph Maina

**Affiliations:** 1 School of International Development, University of East Anglia, Norwich, United Kingdom; 2 Stockholm Resilience Centre, Stockholm University, Stockholm, Sweden; 3 ARC Centre of Excellence for Coral Reef Studies, James Cook University, Townsville, Australia; 4 Marine Programs, Wildlife Conservation Society, Bronx, New York, United States of America; 5 School of Marine Science and Technology, Newcastle University, Newcastle upon Tyne, United Kingdom; 6 Computational Ecology Group, Department of Biological Sciences, Macquarie University, Sydney, Australia; University of Western Australia, Australia

## Abstract

Globally, fisheries are challenged by the combined impacts of overfishing, degradation of ecosystems and impacts of climate change, while fisheries livelihoods are further pressured by conservation policy imperatives. Fishers' adaptive responses to these pressures, such as exiting from a fishery to pursue alternative livelihoods, determine their own vulnerability, as well as the potential for reducing fishing effort and sustaining fisheries. The willingness and ability to make particular adaptations in response to change, such as exiting from a declining fishery, is influenced by economic, cultural and institutional factors operating at scales from individual fishers to national economies. Previous studies of exit from fisheries at single or few sites, offer limited insight into the relative importance of individual and larger-scale social and economic factors. We asked 599 fishers how they would respond to hypothetical scenarios of catch declines in 28 sites in five western Indian Ocean countries. We investigated how socioeconomic variables at the individual-, household- and site-scale affected whether they would exit fisheries. Site-level factors had the greatest influence on readiness to exit, but these relationships were contrary to common predictions. Specifically, higher levels of infrastructure development and economic vitality - expected to promote exit from fisheries - were associated with less readiness to exit. This may be due to site level histories of exit from fisheries, greater specialisation of fishing households, or higher rewards from fishing in more economically developed sites due to technology, market access, catch value and government subsidies. At the individual and household scale, fishers from households with more livelihood activities, and fishers with lower catch value were more willing to exit. These results demonstrate empirically how adaptive responses to change are influenced by factors at multiple scales, and highlight the importance of understanding natural resource-based livelihoods in the context of the wider economy and society.

## Introduction

Globally, many fisheries suffer from an excess of fishing effort endangering their long-term sustainability [1], [2]. Excess fishing effort is particularly difficult to address in small-scale, developing-country fisheries where there are limited options for controlling access [3], [4]. Fishers' livelihoods are also increasingly vulnerable to effects of climate change [5] and conservation initiatives, for example marine protected areas that exclude or restrict resource extraction [6]. The readiness of fishers to exit fisheries and adopt alternative livelihoods is therefore important for the long term sustainability of overexploited stocks, as well as for fishers' ability to adapt to change and manage their vulnerability to displacement from fisheries.

Models based on economic rationality predict that entry to and exit from fisheries are driven by the profitability of fishing [7], [8]. Empirical studies have, however, shown that fishers may be reluctant to exit even when it seems economically rational. This is linked to various cognitive, cultural, and socio-economic factors [9]. Studies of other sectors, such as farming, also highlight the important role of occupational attachment and identity [10]. Characteristics such as age, education and ethnicity have been statistically significant predictors of exit behaviour in some studies [11]–[13], while, cognitive and attitudinal factors such as job satisfaction [14], family traditions [15], occupational attachment and identity [16], [17] and expectations of potential windfalls [18] can also make fishers reluctant to exit. The type of fishing also influences labour mobility due to differences in costs, hazard, accessibility and attractiveness to different people [19], while sunk and fixed capital invested in boats or expensive gears can reduce likelihood of exiting [12], [20]. Exit decisions may also be affected by the function of fishing within a household livelihood. For example, where fishing is a supplementary, risk-spreading activity, exit is expected to be relatively unaffected by economic factors such as opportunity costs [19].

Studies of fishery exit at multiple sites have found considerable variation in readiness to exit between villages and countries [11], [13], [14], suggesting that it is strongly affected by local context, and highlighting the importance of studying effects at different scales. This context dependence is often linked to the availability of alternative livelihoods within the local economy (e.g. [14], [21]). Where alternative livelihoods are available, the opportunity costs of investing labour in fishing are higher, reducing the profitability of, and facilitating exit from fisheries [8]. Economically poorer regions with few attractive alternative livelihoods are therefore predicted to suffer more from overfishing. Recent studies have lent some support to this view, finding higher fish biomass densities near sites with greater infrastructure, and attributing this to an economy less reliant on natural resource extraction [22], [23]. Development of alternative livelihoods has also been shown in some cases to lead to lower levels of fishing effort [18]. In general however, relationships between economic development and dynamics of fishing pressure have rarely been explicitly and empirically tested. In fact small-scale fisheries research has been critiqued for failing to account for fisheries' position within a wider economy [19], [24] or to include a range of contexts that allow for statistical testing, and evidence-based recommendations [3].

Environmental factors, such as lower stocks and catch rates may also explain differences in occupational mobility between sites [20], [25] with fishers who perceive a decline in fisheries more willing to exit [10]. Meanwhile, livelihood strategies are also affected by institutional factors such as welfare support, access rules and subsidies [19]. In summary, exit from fisheries is affected by a wide range of factors including but not restricted to micro-economic factors (Table S1). A predictive understanding of exit behaviour requires consideration of the broader societal and economic context in which decisions are made.

While previous studies have identified a range of individual characteristics and economic factors that influence fishers' decisions to exit a fishery, this is the first to explicitly evaluate how readiness to exit small-scale fisheries is affected by factors operating at different scales. We ask which factors at which scale determine readiness to exit across a gradient of economic development in five western Indian Ocean (WIO) countries. This can help to identify appropriate points of intervention for policies that aim to reduce fishing effort or improve adaptive capacity within fishing communities. The research also contributes to the understanding of the complex and multi-scale nature of adaptive capacity, where non-asset based factors, particularly those concerned with identity, place and culture are increasingly recognized as important factors in decisions and adaptation [26]–[28].

## Methods

### Data collection

**Figure 1:**
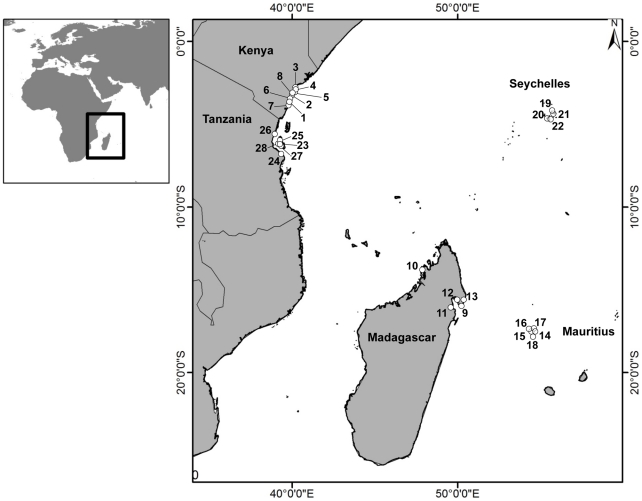
Locations of study sites. Numbers correspond to site numbers in Table S2.

We collected data on individual, household and community characteristics alongside readiness to exit in 28 coastal sites in Kenya, Tanzania, Seychelles, Mauritius, and Madagascar (Table S2, Figure 1) in 2005. Sites included a single community, or group of neighbouring small communities, and were selected purposively from communities that had some dependence on coral reef fisheries, to provide a spectrum of socioeconomic conditions both within and among countries. There is also considerable variability in the state of fish, fisheries resources and wealth in the region, which provides useful contrasts for evaluating potential multi-scale effects [29], [30].

Sampling of households within sites was based on a systematic design, where a fraction of every i^th^ household (e.g. 2^nd^, 3^rd^, 4^th^) was determined by dividing the total population by the sample size [31]. Where the density of fishing households was low, supplementary targeted interviews were held with fishing households based on random selection from lists of fishers. We conducted between 23 and143 surveys per site, depending on the population size.

We conducted household surveys with heads of household or an available adult household member from 1732 households, which included 599 fishers. The survey covered household demographics and livelihood activities, perceptions about resource conditions, any occupational changes experienced by the interviewee in the past five years and specific additional questions about fishing activities if the interviewee was a fisher. Fishers were asked to describe the magnitude of a ‘good’, ‘poor’ and ‘typical’ catch from a day's fishing. We used the typical-day figure to construct hypothetical scenarios involving a reduction of catch. Fishers were asked what they would do in response to a sustained decline in their normal catch of 10%, 20%, 30%, and 50% and responses were coded for each level according to whether fishers envisaged that they would keep fishing or exit the fishery.

### Ethics Statement

We obtained verbal consent from participants before conducting household surveys. During verbal consent, participants were informed about the survey, its purpose, and how the data would be utilized. Participant's names were not recorded. Written consent from participants was not obtained because of low literacy rates in many of our field sites, which meant that participants may not have fully understood what they signed. This project was administered by the Wildlife Conservation Society, which does not have an Institutional Review Board for research ethics regarding social science surveys.

### Selection and measurement of variables

Previous literature identified a number of factors that may influence labour mobility in small-scale fisheries including individual factors, relationship to and perceptions of fishing, livelihoods, fishing characteristics, resource availability and socioeconomic context (Table S1). We selected variables from each of these themes based on a) theoretical or previously empirically demonstrated relationships with fishery livelihoods and labour mobility from the literature, b) reliable measurements of relevant factors within the survey dataset, c) sufficient variation within the data to provide contrast for statistical analysis, and d) lack of collinearity with other variables (Table 1).

**Table 1:** Factors highlighted as affecting fishery exit decisions in the literature, and corresponding variables used in this study.

Scale	Factor	References	Variable used in this study
Individual	Age	[11]–[15], [22]	Age of fisher
	Education	[11]–[15], [22]	Years of formal education
	Experience of/access to other occupations	[13]–[15], [19], [21], [22]	Number of occupations of fisher
Individual Fishing characteristics	Family tradition of fisheries	[15], [21]	Reason given for starting fishing included family or tradition?
	Fishing Experience	[11], [21], [25]	Number of years of fishing experience
	**Type of fishing**	[**19**]	**Type of fishing gear used**
	Capital investment/Vessel owner	[12], [14], [15], [19]–[22]	Ownership of a boat or capital intensive gear
	Catch rate	[21], [22]	Value of normal days catch (ppp)
	Perceived catch rate trend	[11]	Whether fisher perceives decline in fisheries
Household livelihoods and economy	Wealth	[11], [22]	PCA of household characteristics and appliances across entire sample
			PCA of household appliances calculated for each country
	Household occupational structure	[13]–[15], [19], [21], [22]	Number of occupations in the household (in additional to the fishers')
	Role fishing within household livelihood	[14], [19], [22], [32]	Whether fisheries is the top-ranked livelihood activity in the household
Site	Location	[11], [13], [14]	Country
			Site
	Resource abundance	[20], [25]	Biomass density of fishes on nearby reefs
	Local economy	[12], [13], [15], [15], [19,21]	Proportion of interviewees in the community who had changed occupation in previous 5 years and preferred their new occupation
			Proportion of households in the community who's primary livelihood is fisheries
	Socioeconomic development and isolation	[13], [15], [15,19]	Factor analysis of presence of 16 infrastructure items

#### Individual- and household-level factors

Survey questions facilitated collection of demographic information on age, education and experience of other occupations. Wealth was evaluated by using a Material Style of Life (MSL) scale based on the presence or absence of various household possessions such as a TV, a toilet, radio, and the type of material the house was constructed from, factor analyzed using principal component analysis in SPSS [32]. A ‘global’ MSL scale was calculated from the full sample of households to represent absolute levels of wealth across the entire region. This global MSL scale was calculated as the first principal component of the presence of 15 material assets that were relevant across all countries: vehicle, electricity, television, gas or electric stove, fan, piped water, refrigerator, radio, video player, and the type of walls, roof, and floors (see [30] for further details). In addition, we developed a more sensitive, national MSL score to allow greater resolution in differentiating wealth status between households within each country. This national MSL was based on factor analysis of the most appropriate wealth indicators in each country (Table 2). Some variables that were strong wealth indicators in one country had little or no variation in other countries (e.g. none of the respondents in Madagascar had tile floors, but this was important in distinguishing wealthier from poorer households in Seychelles). The variance explained by the National MSL scores was 57.4% for Kenya; 40.7% for Madagascar; 45.1% for Tanzania; 40.5% for Mauritius; and 33.2% for Seychelles.

**Table 2:** Factor loadings of country-level material style of life scores.

Indicator	Kenya	Tanzania	Mauritius	Seychelles	Madagascar
Electricity		0.58			
Fan		0.45	0.67		
Floor: cement	0.88	0.84			
Floor: dirt/bush material	−0.89	−0.85			−0.78
Floor: tile				0.22	
Generator			0.52		
Mattress					0.31
Radio	0.27	0.27			0.49
Roof: metal	0.78	0.79		−0.72	0.81
Roof: thatch	−0.80	−0.79			
Roof: tile				0.72	
Satellite			0.71		
Toilet: flush	0.35		0.53		
Toilet: none		−0.40			−0.42
Toilet: outhouse				−0.56	
TV		0.49			
VCR/Video machine			0.61	0.42	
Vehicle			0.74	0.02	
Wall: bamboo	−0.88	−0.81			
Wall: cement		0.78		0.76	
Wall: metal				−0.75	
Wall: stone or concrete	0.89				
Wall: wooden plank					0.81
Water Tank			0.65	0.54	

#### Fishery factors

We characterised each fisher according to their principal fishing gear used and recorded whether they had made a significant capital investment in the fishery, in terms of a boat or ‘capital intensive gear’ (e.g. large net) as judged according to the local fishery context in which they operated. We asked fishers why they started fishing and coded their responses into tradition, necessity, or free choice. We asked fishers about their perception of the condition of the fishery resource compared to five years previously, and coded responses into a binary variable indicating whether they perceived a decline. To account for different values of different species, catch rate was calculated as reported catch value in US$ adjusted for national purchasing price parity.

#### Site-level factors

We used the full sample of random household surveys – fishers and non-fishers - in each community to calculate the proportion of households involved in fisheries and the proportion of interviewees who had changed occupation within the previous five years and preferred their new occupation. The latter was designed to indicate whether the local economy was providing attractive alternative occupations that may affect the opportunity cost of labour.

We developed an ‘infrastructure index’ for each site, as described in [33]. This index was based on the presence of 20 infrastructure items [34] including: hospital, medical clinic, doctor, dentist, primary school, secondary school, piped water, sewer, sewage treatment, septic tanks, electricity service, phone service, food market, pharmacy, hotel, restaurant, petrol station, public transportation, paved road, banking facilities. We ran Factor Analysis on the presence or absence of these infrastructure items and used the first principal component as the infrastructure index, socio-economic development and isolation.

Biomass of reef fishes (kg/ha) on adjacent reefs was taken as an indicator of the condition of local fisheries resources. Reef fish biomass has been found to be a sensitive indicator of fishing pressure in the region [33], [35]. Biomass was recorded by underwater visual census by two experienced observers (T.R. McClanahan and N.A.J. Graham) and estimated by length-weight relationships for species or families) as described in [33].

### Analysis

Following visual assessment of responses to 10, 20, 30 and 50% declines, we focussed on the response to a 50% decline for detailed analysis as this provided approximately equal proportions of fishers who stated they would exit or remain fishing. We used three types of analysis to identify site, household, and individual-level factors predicting fishers' stated response to a 50% decline.

Firstly, we used classification trees to compare the predictive ability of factors at individual, household, and site level. Univariate tree models are an improvement on conventional linear and logistic regression methods where non-linear relationships and multiple interactions between predictor variables are likely, because they are non-linear and do not require the *a priori* specified interaction terms in the model [36]. In our multi-scale dataset we expected interactions between factors at different scales. For example the importance of wealth might be greater in some countries than others. Classification trees could account for this in a straightforward way by including wealth variables in lower branches of the tree after classification by country or site. We ran classification tree analysis using site as a nominal variable to compare the predictive ability of individual and household-level factors compared to the site a fisher was living in. We also ran the analysis without site, but including site-level variables to check the ability of variables at site level to classify responses compared to individual and household variables and country (if site-level variables and site name are both included in a classification tree analysis, site name will always be selected in preference to any variables measured at the site scale).

Secondly, we examined the relationship between readiness to exit and site-level variables. For this analysis, we used regression trees and bivariate plots to examine the community-scale variables that predicted the proportion of fishers opting to exit at each site.

Thirdly, we assessed whether individual or household-level variables predicted variation in responses, beyond the variation explained by site. We fitted a generalised linear mixed model (GLMM) to assess the ability of these variables to predict individual stay or exit responses, with site a-priori set as a random intercept to account for inter-site variation. We assessed the full model for the significance of individual variables, and then ran a stepwise selection based on AIC to remove or add variables to find the most parsimonious model. The GLMM analysis was preceded by analysis of collinearity by calculating variance inflation factors [36]. Household-level occupational multiplicity was replaced with the number of different occupations in the household in addition to individual respondents' occupations, in order to reduce collinearity between individual and household-level occupational multiplicity, and to clarify their relative contributions. Occupational multiplicity variables and the normal catch value were log-transformed before GLMM analysis to provide a more normal distribution. All statistics were conducted with the rpart, glmmML and design packages in R [37].

## Results

**Figure 2:**
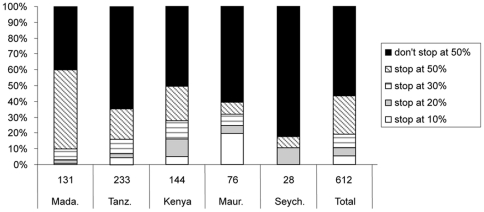
Thresholds for exiting the fishery in each studied country. Sample size in each country give beneath the bar. Mada. = Madagascar, TZ = Tanz., Maur. = Mauritius, Seych = Seychelles.

Greater proportions of fishers responded that they would exit fisheries in response to higher levels of hypothetical decline (Figure 2). At 50% reduction in catch just under half of the 599 fishers reported that they would stop fishing although the proportions of fishers exiting varied between countries from 19% in Seychelles to 60% in Madagascar.

### Multi-scale analysis

**Figure 3:**
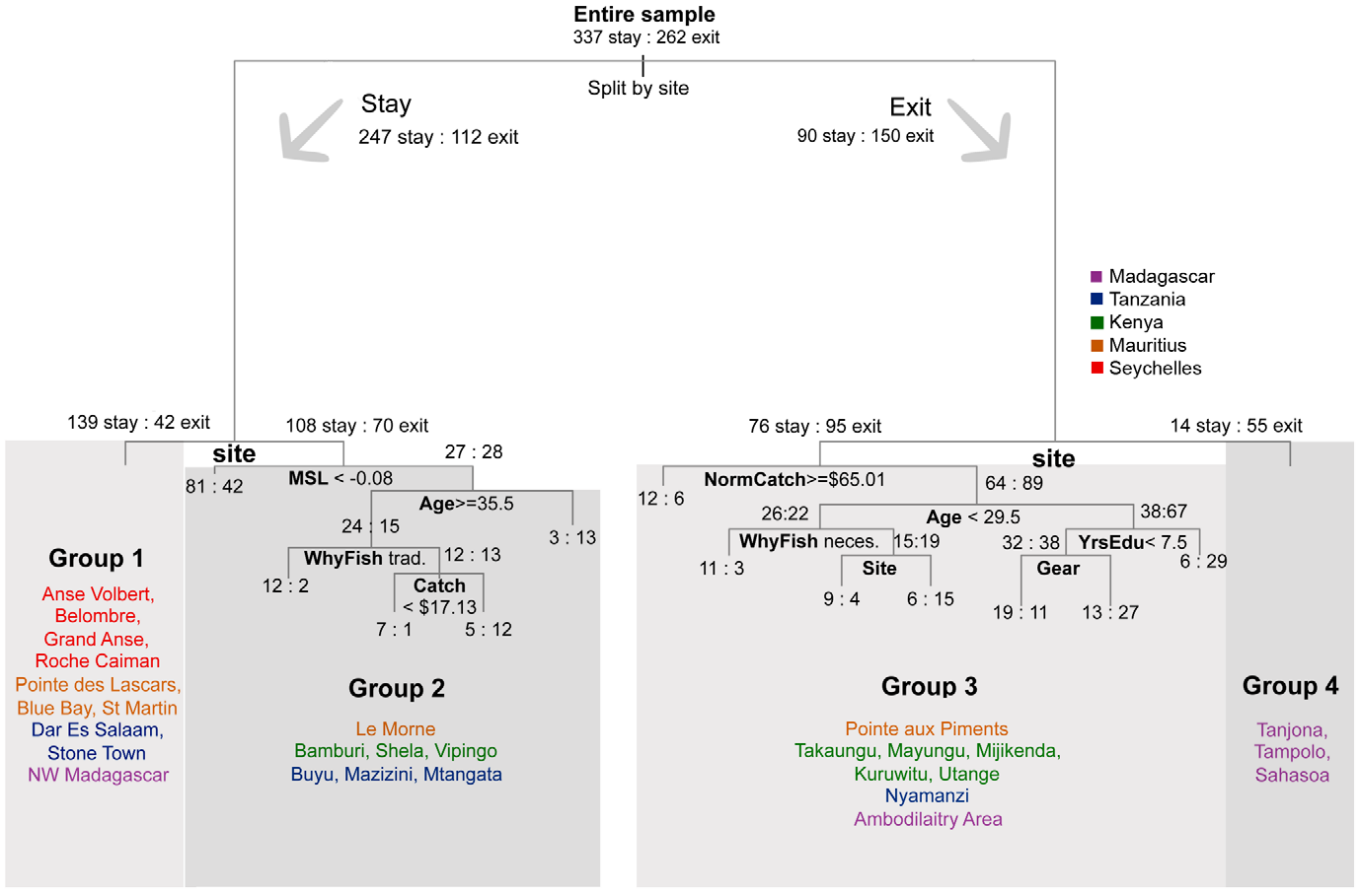
Classification-tree analysis evaluating stay and exit decisions in the studied fisheries in response to a hypothetical halving of catch value. Based on the responses of 599 Western Indian Ocean fishers. Splits were based potentially on all variables described in Table 1. Sites are coloured by country (Blue – Seychelles, Green – Mauritius, Purple – Tanzania, Black – Kenya, Red – Madagascar). Numbers of fishers opting to stay (left) and exit (right) are shown at each branch. Fishers meeting the split conditions [e.g. material style of life (MSL)<−0.08] pass down to the next left-hand branch.

Classification-tree analysis identified the variable ‘site’ as having the greatest power to predict readiness to exit (Figure 3), which was also better for predicting effort than individual- or household-level factors. The nominal variable for ‘site’ was responsible for the first three splits (dividing fishers by four different groups of sites) before other variables were incorporated lower in the tree. Financial factors, including material style of life and catch value, divided fishers in Groups 2 and 3, dominated by Kenyan and Tanzanian sites. Wealthier fishers from Group 2 were more likely to exit, while fishers with higher catch value in Group 3 were less likely to exit. Individual variables (age, education, why they started fishing and used gears) appeared lower in the tree to separate groups 3 and 4. Age, education and catch value had contradictory effects in different branches of the tree suggesting context-dependent interactions between factors. Regression tree splits using stated reason to start fishing indicated that fishers who cited tradition and free choice were less likely to opt for exit. Classification-tree analysis without the site variable used site-level biomass to split off fishers from the three Madagascar sites with high biomass and % exit, then mostly used individual and household factors (Figure S1) suggesting that the predictive power of site was not fully reflected in any of site-level variables (e.g. infrastructure) included in this analysis.

### Site-scale factors

**Figure 4:**
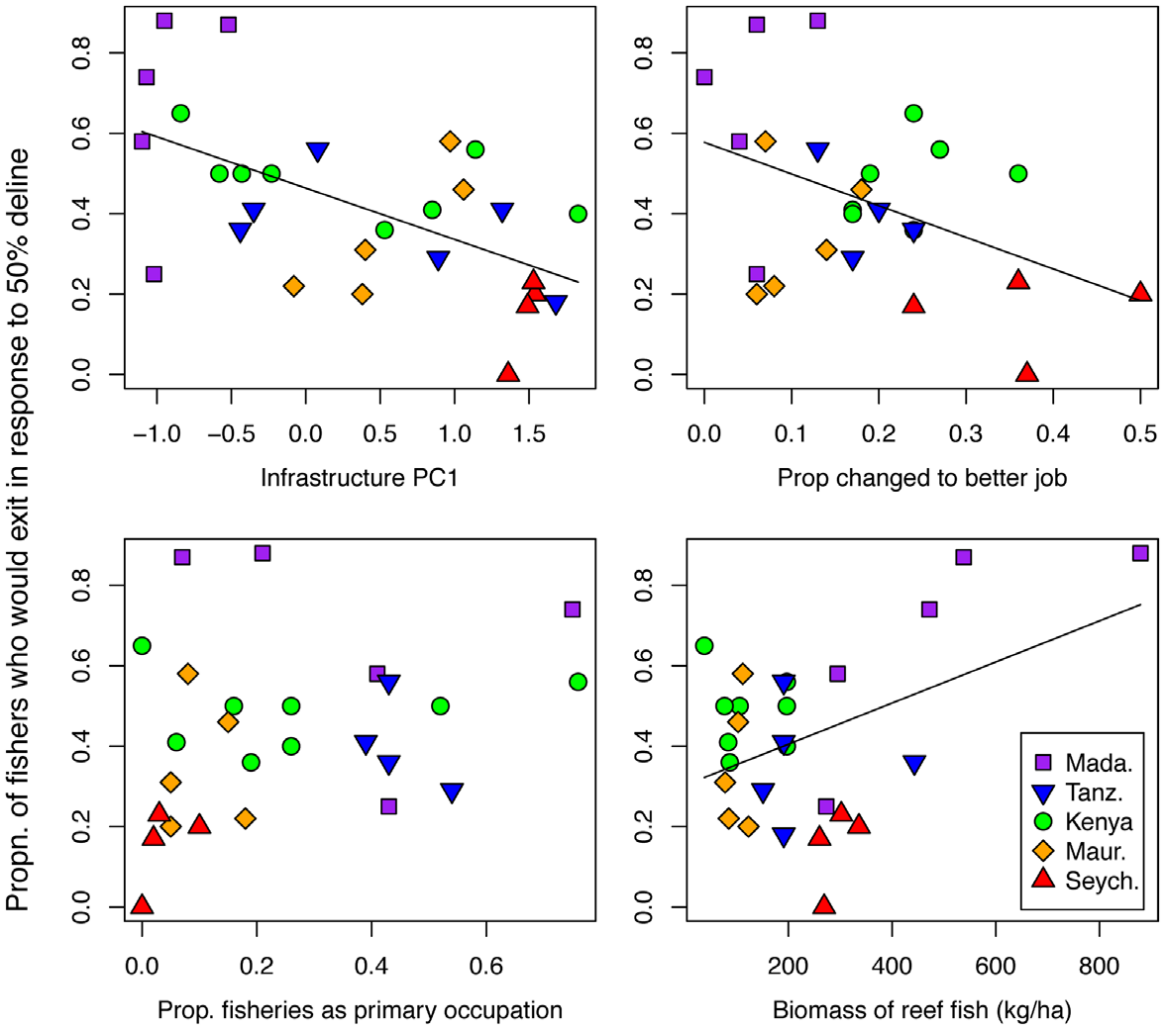
Relationship between percentage of fishers at a site who would exit in response to a 50% catch decline and site-scale variables. Lines indicate significant relationships (p<0.05).

Given the importance of site for predicting willingness to exit, we examined how site-scale factors were related to the proportion of fishers at that site who would exit the fishery. Regression tree analysis resulted in splits according to the infrastructure index which separated low infrastructure sites (dominated by Madagascar) with high proportions of fishers exiting. A second split also used infrastructure, and split off a group of high infrastructure sites, dominated by Seychelles, with low proportions of fishers exiting (Figure S2). This negative infrastructure-exit relationship was also reflected in a significant negative linear relationship between infrastructure and proportion of fishers exiting across the region (β = −0.129, p = 0.003, Fig. 4a). Although there were some trends between countries, with Madagascar and Seychelles sites tending to have high and low percentage exiting respectively, the infrastructure variable was selected by the regression tree in preference to the term ‘country’.

The proportion of household surveys reporting a favourable change in occupation in the previous five years was weakly to moderately correlated with infrastructure (Spearman's Rho = 0.499), and also showed a significant negative linear relationship with proportion of fishers willing to exit (β = −0.788, p = 0.027, Figure 4b). These negative relationships across the region were not evident among sites within individual countries. Within individual countries, the only relationships between infrastructure and percentage exiting were in fact positive (Seychelles: β = 1.223, p = 0.015, Mauritius, β = 0.301, p = 0.055).

The proportions of households within a site with fishing as a primary occupation was not significantly related to the percent exiting (p = 0.150, Figure 4c). Biomass of fish on adjacent reefs was positively related to percentage exiting (p = 0.028), but this relationship was driven by high biomass values at three Madagascar sites (Figure 4d).

### Household and individual scale analysis

When the effect of site was accounted for by a random effect, only two of the sub-site scale variables were significant in the full GLMM model. Both occupational multiplicity at the household scale, (i.e. the number of occupations additional to the fishers') (p = 0.017) and typical daily catch value (p<0.001), were positively related to exit (Table S4). This was further supported by the stepwise selection of the GLMM, which resulted in only these two fixed effect factors being maintained (Figure S3).

## Discussion

As with the literature, a wide range of economic and non-economic variables measured at different scales were related to readiness to exit fisheries. Although responses differed between countries, with high levels of readiness to exit in Madagascar and low levels in Seychelles, site-level differences were most prominent. Beyond the strong effect of site, we also found that household and individual characteristics also influenced fishers' willingness to exit. Individual fishers whose fellow householders had more occupations and whose catches were larger were less willing to exit.

### Relationships between exit and site-level factors

In agreement with previous work this study found that the readiness to exit a fishery varied significantly between sites [10], [12], [13]. Site-level variation in responses may be due to differences between individuals in different sites, for example one community may have wealthier households than another. Alternatively, site differences may be due to site-level factors, such as the economic context or local geography, which influence responses to decline independently of the variation between individuals. Without large, multi-scale studies it has been difficult to untangle the scale at which adaptive response of fishers is primarily determined. This study strengthens distinction between individual, household, or site-level factors in influencing fisher's decisions to exit fisheries and provides strong support for the importance of site-level factors. The local social and cultural context appears to play a large role in fishers' perceived willingness to exit the fishery. Place-based factors have similarly been found important in determining adaptation and resilience in a range of risk settings [38], [39]. In particular, sites with lower levels of infrastructure, and less evidence of favourable occupational mobility had higher proportions of fishers who would exit.

The direction of relationships between site-scale variables and fishery exit were surprising and contrary to conventional understanding of fishery bioeconomics.

The site-level variables are indicative of a broadly defined ‘economic development’. The infrastructure index indicates both material community development as well as the degree of connectedness to global and national economies. The proportion of households whose members had favourably changed jobs within the previous five years, and the proportion of households with fishing as a primary occupation are indicators of the nature of employment opportunities available in the local economy.

Smith et al. [19] identify the importance of the non-farm economy being either “a residual sector offering only coping activities and absorbing labour displaced from traditional activities of farming and fishing etc., or a dynamic one creating new jobs, exerting upward pressure on wages”. Indicators of the vibrancy of the local economy would be expected to accompany more alternative livelihood opportunities for fishers, increased opportunity costs of labour and higher readiness to exit [8], [19], [40]. In contrast, we found the proportion of households where members had favourably changed jobs within the previous five years was negatively related to the proportion of fishers willing to exit. In summary, levels of economic development, broadly defined were negatively correlated with exit when the reverse would be expected.

We suggest three plausible and possibly complementary explanations for these results:

- Levels of economic development and diversification, indicated by the infrastructure index and occupational mobility variable may be associated with specialisation of livelihoods and professionalization of employment, including fisheries. In regions and countries with better infrastructure and more diversified economies, fisheries are more specialised and professionalised and less frequently part of multiple household livelihoods [23]. Specialised fishers may be well vested and have lower capacity to diversify employment as an adaptation [16], [17].
- Fishers in more economically developed sites may be more committed due to the history of change in fisheries during the process of development. Fishers at developed sites have chosen fisheries in the context of other available occupations. In contrast, less economically developed sites with fewer opportunities have higher proportions of fishers, many of whom pursue a diverse livelihood strategy [23], [24] and may be willing to exit if alternatives were available. Thus, as local economies develop and provide alternative occupations, less committed fishers may exit and take up other occupations, leaving a smaller, but more specialized and ‘hard-core’ population of fishers, resulting in the negative relationship between readiness to exit and level of economic development observed here. Our cross-site comparative snapshot methodology does not capture the dynamics of change over time as contextual factors evolve, and studies over time would allow for a better evaluation of this hypothesis.
- Markets, technology and government assistance facilitate and reward fishing more in economically developed sites. The value of the catch varied greatly between countries, being for example two orders of magnitude greater in Seychelles than Madagascar (Table S3), despite the high biomass densities in some Madagascar sites. While, some studies in developed countries have found that abundance of fishery resources can lead to lower occupational mobility [20], [25] the high biomass in Madagascar sites was associated with a high readiness to exit (Fig. 3d). We suggest this is an association with development and pricing where economic development and connectivity promote higher prices for these resources. Thus, despite abundant reef resources in some sites in Madagascar, technology, specialisation, catch volumes and market access allowed higher catch revenues in more developed areas, which may reduce the willingness to exit. Similar trends have been reported for bushmeat, where lower supplies are related to higher value across rural-urban gradients in Africa [41].

Government support may also influence fisheries behaviours more in developed locations through subsidies and infrastructure. For example in Seychelles, fishers are supported by government funded fuel subsidies and health insurance, while in Mauritius registered fishers receive a bad weather allowance, which compensates them when fishing activity is disrupted by unfavourable weather.

A simplistic perspective in which poverty, lack of alternative occupations and overexploitation are mutually reinforcing (see [42]) predicts that economic development supports fishers' occupational mobility by increasing opportunity costs of labour. But if development also results in specialisation of livelihoods, increased profitability, and greater institutional support for the industry, the readiness of fishers to exit may decrease. This view is supported by the observed fidelity of fisheries labour in relatively wealthy western nations [9], [14]. Low mobility of fishing labour has largely been attributed to poverty and a lack of livelihood alternatives that is associated with economic underdevelopment [40]. Yet, in our case study, the richness of rewards may also lead to high fidelity especially in contexts of market access, technology, infrastructure, and government subsidy.

Interestingly, the unexpected direction of relationships between economic development and readiness to exit was apparent only at the largest scale. The negative relationship between readiness to exit and infrastructure across the region was not apparent within individual countries. Instead, the only significant within-country relationships between exit and infrastructure, in the Seychelles and Mauritius were positive. Consequently, the direction of the relationship may differ with the scale of analysis because of different processes operating at different scales. For example, the differences in infrastructure within a small island country may improve alternative livelihood options, while the larger differences observed across the region may be associated with structural differences in national economies and fisheries policy, leading to the large-scale negative relationship.

### Household and individual-scale influences on exit

The study also found that household–scale factors affected readiness to exit as reported in Kenya, where household occupational multiplicity was positively related to readiness to exit [22]. This study extends this result in two key ways. Firstly, we find that the positive relationship between household occupational multiplicity and exit exists over a range of countries. Secondly, we identified that this relationship operates at the household- rather than either individual or community scales. Specifically, the multiplicity of additional household occupations rather than the fishers' own occupational multiplicity was the significant variable, and this persisted after accounting for between-site variability. Fishers may benefit from the support of their fellow household members if they have to stop fishing due to fishery declines. Conversely, where fishers are the only source of livelihood, they may need to continue fishing to support their household regardless of declines.

This suggests that adaptation of fishers to disturbances or fisheries declines, and reduction of fishing effort, may be facilitated by alternative livelihoods, even if they are not suitable to fishers themselves. This contrasts with the assertion concerning seaweed farming, often conducted by women, that extra household occupations may subsidise rather than reduce fishing activity [43]. Complex household decisions require further empirical study at the household scale but it seems clear that the overall household livelihood portfolio is relevant to fisher decisions and willingness to exit [24]. Household member occupations may subsidise continued fishing or exiting from the fisheries depending on other contextual variables such as job choices and cultural affinity to fishing. The lack of relationship with the individual's occupational multiplicity is also surprising. This may be due to part-time fishers, who on one hand have other options and could stop fishing but, on the other, do not rely on fisheries, and so can absorb declines without exiting.

Household wealth was found to positively affect the willingness to exit in Kenya [22]. This was broadly supported here by the classification tree analysis in which absolute material style of life appeared within one branch of the tree for the group 2 sites, dominated by Kenya and Tanzania, where fishers from wealthier households were more likely to exit. It was not a significant factor, however, when examined relative to other households within the community and accounting for community-scale effects. Thus wealth may be an important factor at higher scales rather than affecting exit decisions of fishers within their community. Alternatively, household wealth was collinear to catch value, a stronger indicator in this study that may have masked the household wealth effect.

Normal daily catch value was the strongest determining factor at the individual scale, and was negatively related to readiness to exit. This makes practical sense, as we asked about a catch decline proportional to stated normal catch. Fishers with higher value catches may have more scope to absorb declines. Different results may have been found by comparing responses to absolute as well as relative declines [44], although a full standardization of the effect of absolute declines across the wide range of gears, species and contexts in this region would be challenging. The result also agrees with observations from decommissioning and microeconomics, that less successful fishers have greater incentives to exit due to the lower value of their labour within the fishery [45]. This has implications for the dynamics of the fishery system and recovery of overexploited fisheries. If the least successful fishers preferentially exit, the decline in fishing mortality will be less than predicted by the numbers of fishers exiting.

Several of the characteristics we looked at were poor predictors of readiness to exit. Individual-scale characteristics identified in other studies [11]–[14], [20] as influencing occupational mobility were not significant. Age and education were identified on lower branches of the classification tree but responded differently in two branches. The stated reason for starting to fish, an indicator of cultural links or the personal appeal of fisheries as an occupation, appeared in two lower branches of the classification tree, suggesting limited local relevance. Further, the ownership of capital-intensive gears had no detectible effect in this study. One possible explanation is that patron-client arrangements with middlemen, common in the region, may reduce readiness to exit in non-gear owners due to credit arrangements that may encourage fishing in times of poor catches [46].

### Relevance to adaptive capacity

Climate change, globalisation and environmental degradation are leading to unprecedented levels of change and disturbance to social-ecological systems such as fisheries, and their associated livelihoods. Our study provided further evidence that adaptation is influenced by multiple scales that go beyond the assets and characteristics of individuals and include the social and economic environment enabling adaptation [47], [48]. Our study, using hypothetical questions within the context of a livelihood activity provides a means to empirically test determinants of an element of adaptive capacity over a large scale and range of contexts, in response to incremental environmental change rather than extreme events. The results do not support the widespread belief and policy theme that the poor are less able to adapt than the wealthy. Rather these findings add to growing literature which identifies multiple interlocking and dynamic factors which make up adaptive capacity, and specifically emerging insights into existing or shifting livelihood as an adaptive response.

### Conclusions

Fishers face an increasing variety of changing conditions related to overexploitation, climate change, globalization, and conservation of marine biodiversity. Understanding how fishers will respond to these ecosystem and institutional changes is critical to better managing fisheries and improving the livelihoods of those dependent on fisheries. Some conventional fisheries economic thinking was supported by this large-scale study, such as the greater occupational mobility of less-successful fishers, while some was refuted. In particular, the often-assumed positive relationship between economic development, and mobility of fishing labour was contradicted by our results. Our results highlight the strong context dependency in adaptive responses, requiring different recommendations and interventions in different contextual conditions.

## Supporting Information

Figure S1 **Classification-tree analysis for the responses of 599 fishers to hypothetical declines including site-level variables, but not including the variable of ‘site’.** Branches classifying fishers as ‘exit’ are on the right hand branches.
(DOCX)

Figure S2 **Regression-tree analysis for proportion of fishers within a site opting to stop fishing in response to a halving of catch value.**
(DOCX)

Figure S3 **Relationships between the probability of a fisher stating they would exit from a hypothetical 50% decline in catch and the occupational multiplicity of their fellow householders, the value of their typical daily catch.**
(DOCX)

Table S1 **Factors considered in previous empirical studies of fisher mobility and whether they were found to be significant (if statistically tested).**
(DOCX)

Table S2 **Communities surveyed and summary statistics for each.** * Only targeted fisher surveys (no random household surveys) conducted in Stone Town or Mazizini.
(DOCX)

Table S3 **Variables included in analysis and data summarised by country.**
(DOCX)

Table S4 **Results of GLMM regression with all individual or household variables.**
(DOCX)

## References

[1] World Bank, FAO (2009). The sunken billions..

[2] Worm B, Hilborn R, Baum JK, Branch TA, Collie JS (2009). Rebuilding Global Fisheries.. Science.

[3] Pauly D (2006). Major trends in small-scale marine fisheries, with emphasis on developing countries, and some implications for the social sciences.. Maritime Studies.

[4] Salayo N, Garces L, Pido M, Viswanathan K, Pomeroy R (2008). Managing excess capacity in small-scale fisheries: Perspectives from stakeholders in three Southeast Asian countries.. Marine Policy.

[5] Cheung WWL, Lam VWY, Sarmiento JL, Kearney K, Watson R (2009). Projecting global marine biodiversity impacts under climate change scenarios.. http://dx.doi.org/10.1111/j.1467-2979.2008.00315.x.

[6] Mascia MB, Claus CA (2009). A Property Rights Approach to Understanding Human Displacement from Protected Areas: the Case of Marine Protected Areas.. Conservation Biology.

[7] Gordon HS (1954). The economic theory of a common property resource: the fishery.. Journal of Political Economics.

[8] McManus JW (1997). Tropical marine fisheries and the future of coral reefs: a brief review with emphasis on Southeast Asia.. Coral Reefs.

[9] OECD (2007). Structural Change in Fisheries: Dealing with the Human Dimension..

[10] Marshall NA (2010). Understanding social resilience to climate variability in primary enterprises and industries.. Global Environmental Change.

[11] Pollnac RB, Pomeroy RS, Harkes IHT (2001). Fishery policy and job satisfaction in three southeast Asian fisheries.. Ocean & Coastal Management.

[12] Bailey C (1982). Small-scale fisheries of San Miguel Bay, Philippines occupational and geographic mobility..

[13] Panayotou T, Panayotou D (1986). Occupational and geographical mobility in and out of Thai fisheries.

[14] Pita C, Dickey H, Pierce GJ, Mente E, Theodossiou I (2010). Willingness for Mobility amongst European fishermen.. Journal of Rural Studies.

[15] Terkla DG, Doeringer PB, Moss PI (1988). Widespread labour stickiness in the New England offshore fishing industry: Implication for adjustment and regulation.. Land Economics.

[16] Coulthard S (2008). Adapting to environmental change in artisanal fisheries–Insights from a South Indian Lagoon.. Global Environmental Change.

[17] Marshall NA, Fenton DM, Marshall PA, Sutton SG (2007). How Resource Dependency Can Influence Social Resilience within a Primary Resource Industry.. Rural Sociology.

[18] Hill NAO, Rowcliffe MJ, Koldewey HJ, Milner-Gulland EJ (2011). The Interaction between Seaweed Farming as an Alternative Occupation and Fisher Numbers in the Central Philippines.. Conservation Biology..

[19] Smith LED, Khoa SN, Lorenzen K (2005). Livelihood functions of inland fisheries: Policy implications for developing countries.. Water Policy.

[20] Pradhan NC, Leung PS (2004). Modeling trip choice behavior of the longline fishers in Hawaii.. Fisheries Research.

[21] Ikiara MM, Odink JG (1999). Fisherman's Resistance to Exit Fisheries.. http://dare.uva.nl/record/79936.

[22] Cinner JE, Daw T, McClanahan TR (2009). Socioeconomic Factors that Affect Artisanal Fishers' Readiness to Exit a Declining Fishery.. Conservation Biology.

[23] Cinner JE, Bodin Ö (2010). Livelihood Diversification in Tropical Coastal Communities: A Network-Based Approach to Analyzing “Livelihood Landscapes.”. PLoS ONE.

[24] Allison EH, Ellis F (2001). The livelihoods approach and management of small-scale fisheries.. Marine Policy.

[25] Ward JM, Sutinen JG (1994). Vessel entry-exit behavior in the Gulf of Mexico shrimp fishery.. American Journal of Agricultural Economics.

[26] Grothmann T, Patt A (2005). Adaptive capacity and human cognition: The process of individual adaptation to climate change.. Global Environmental Change.

[27] Blennow K, Persson J (2009). Climate change: Motivation for taking measure to adapt.. Global Environmental Change.

[28] Frank E, Eakin H, López-Carr D (2011). Social identity, perception and motivation in adaptation to climate risk in the coffee sector of Chiapas, Mexico.. Global Environmental Change.

[29] McClanahan TR, Graham NAJ, MacNeil MA, Muthiga NA, Cinner JE (2011). Critical thresholds and tangible targets for ecosystem-based management of coral reef fisheries.. Proceedings of the National Academy of Sciences.

[30] McClanahan TR, Cinner JE, Maina J, Graham NA, Daw TM (2008). Conservation action in a changing climate.. Conservation Letters.

[31] Henry GT (1990). Practical sampling.

[32] Cinner J, Pollnac R (2004). Poverty, Perceptions and Planning: Why socioeconomics matter in the management of Mexican reefs.. Ocean & Coastal Management.

[33] Cinner JE, McClanahan TR, Daw TM, Graham NAJ, Maina J (2009). Linking Social and Ecological Systems to Sustain Coral Reef Fisheries.. Current Biology.

[34] Pollnac, Richard B (1998).

[35] Jennings S, Grandcourt EM, Polunin NVC (1995). The effects of fishing on the diversity, biomass and trophic structure of Seychelles' reef fish communities.. Coral Reefs.

[36] Zuur AF, Ieno EN, Smith GM (2007). Analysing Ecological Data.

[37] R Development Core Team (2008). R: A language and environment for statistical computing.. http://www.R-project.org.

[38] Adger WN, Barnett J, Chapin FS, Ellemor H (2011). This Must Be the Place: Underrepresentation of Identity and Meaning in Climate Change Decision-Making.. Global Environmental Politics.

[39] Cutter SL, Barnes L, Berry M, Burton C, Evans E (2008). A place-based model for understanding community resilience to natural disasters.. Global Environmental Change.

[40] Pauly D (1988). Some definitions of overfishing relevant to coastal zone management in Southeast Asia.. Tropical Coastal Area Management.

[41] Brashares JS, Golden CD, Weinbaum KZ, Barrett CB, Okello GV (2011). Economic and geographic drivers of wildlife consumption in rural Africa.. Proceedings of the National Academy of Sciences.

[42] Bene C (2003). When Fishery Rhymes with Poverty: A First Step Beyond the Old Paradigm on Poverty in Small-Scale Fisheries.. World Development.

[43] Sievanen L, Crawford B, Pollnac R, Lowe C (2005). Weeding through assumptions of livelihood approaches in ICM: Seaweed farming in the Philippines and Indonesia.. Ocean & Coastal Management.

[44] Muallil RN, Geronimo RC, Cleland D, Cabral RB, Doctor MV (2011). Willingness to exit the artisanal fishery as a response to scenarios of declining catch or increasing monetary incentives.. Fisheries Research.

[45] Clark CW, Munro GR, Sumaila UR (2005). Subsidies, buybacks, and sustainable fisheries.. Journal of Environmental Economics and Management.

[46] Crona B, Nyström M, Folke C, Jiddawi N (2010). Middlemen, a critical social-ecological link in coastal communities of Kenya and Zanzibar.. Marine Policy.

[47] Vincent K (2007). Uncertainty in adaptive capacity and the importance of scale.. Global Environmental Change.

[48] Engle NL (2011). Adaptive capacity and its assessment.. Glob Environ Change-Human Policy Dimens.

