# Correction: To Fish or Not to Fish: Factors at Multiple Scales Affecting Artisanal Fishers' Readiness to Exit a Declining Fishery

**DOI:** 10.1371/journal.pone.0172075

**Published:** 2017-02-08

**Authors:** Tim M. Daw, Joshua E. Cinner, Timothy R. McClanahan, Katrina Brown, Selina M. Stead, Nicholas A. J. Graham, Joseph Maina

There is an error in the fourth sentence of the Discussion. The correct sentence is: Individual fishers whose fellow householders had fewer occupations and whose catches were larger were less willing to exit.
