# Supplementary material for: To Fish or Not to Fish: Factors at Multiple Scales Affecting Artisanal Fishers' Readiness to Exit a Declining Fishery
Source: PLoS One. 2012 Feb 10;7(2):e31460. doi: 10.1371/journal.pone.0031460 (PMC3277441; doi:10.1371/journal.pone.0031460)
Supplement: Figure S1 — Classification-tree analysis for the responses of 599 fishers to hypothetical declines including site-level variables, but not including the variable of ‘site’. Branches classifying fishers as ‘exit’ are on the right hand branches. (DOCX) [file pone.0031460.s001.docx]

**Supporting Information**


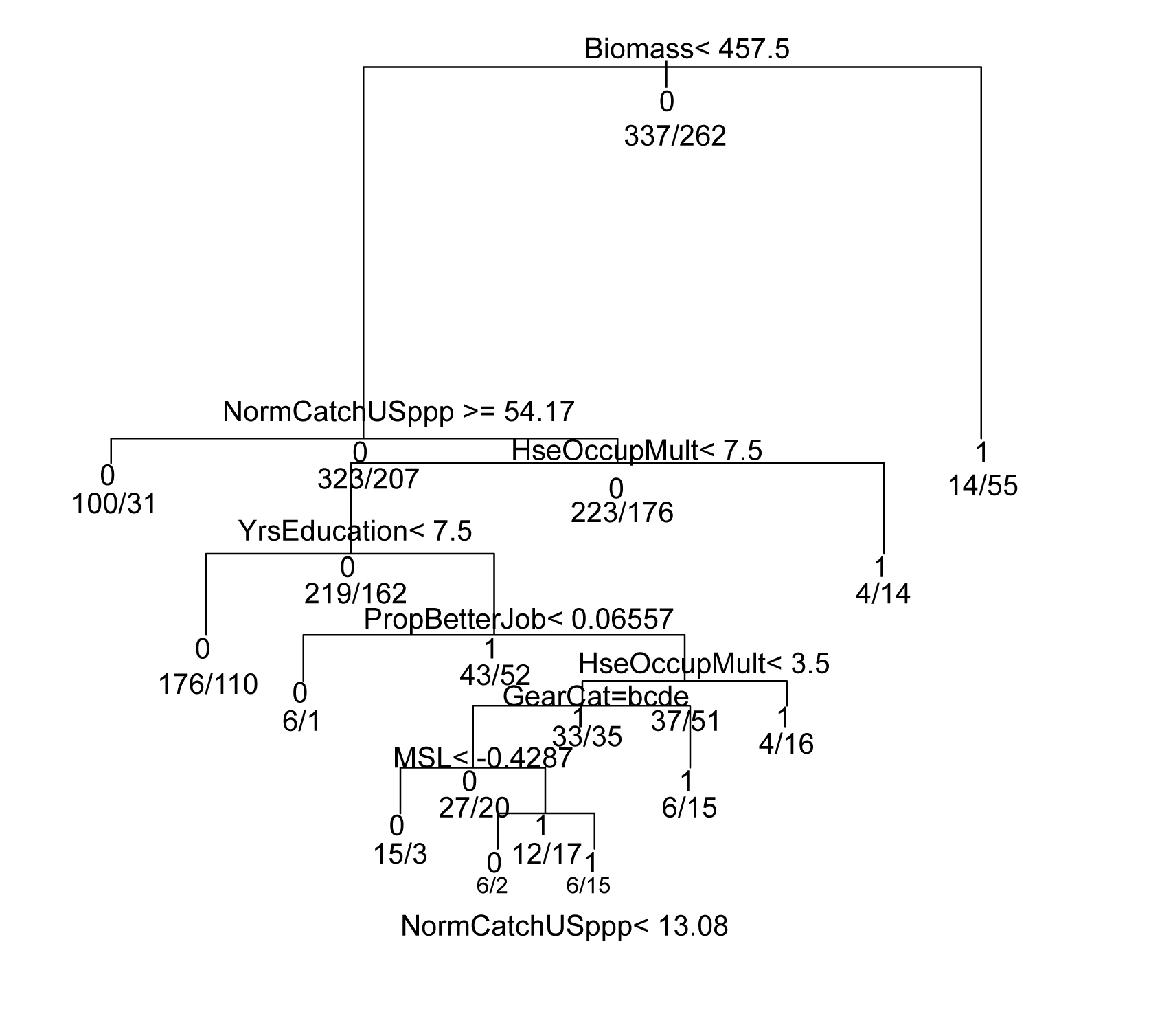


Figure S1. Classification-tree analysis for the responses of 599 fishers to hypothetical declines including site-level variables, but not including the variable of ‘site’. Branches classifying fishers as ‘exit’ are on the right hand branches.
