# Supplementary material for: To Fish or Not to Fish: Factors at Multiple Scales Affecting Artisanal Fishers' Readiness to Exit a Declining Fishery
Source: PLoS One. 2012 Feb 10;7(2):e31460. doi: 10.1371/journal.pone.0031460 (PMC3277441; doi:10.1371/journal.pone.0031460)
Supplement: Figure S2 — Regression-tree analysis for proportion of fishers within a site opting to stop fishing in response to a halving of catch value. (DOCX) [file pone.0031460.s002.docx]

**Supporting Information**

Figure S2. Regression-tree analysis for proportion of fishers within a site opting to stop fishing in response to a halving of catch value. Community-scale variables were offered to the tree including country, infrastructure index, proportion of survey that had favourably changed occupation in previous 5 years, proportion of survey with fisheries as a primary livelihood, biomass of fish on nearby reefs. Proportion of households in the community who's primary livelihood is fisheries
