# Supplementary material for: To Fish or Not to Fish: Factors at Multiple Scales Affecting Artisanal Fishers' Readiness to Exit a Declining Fishery
Source: PLoS One. 2012 Feb 10;7(2):e31460. doi: 10.1371/journal.pone.0031460 (PMC3277441; doi:10.1371/journal.pone.0031460)
Supplement: Figure S3 — Relationships between the probability of a fisher stating they would exit from a hypothetical 50% decline in catch and the occupational multiplicity of their fellow householders, the value of their typical daily catch. (DOCX) [file pone.0031460.s003.docx]

**Supporting Information**


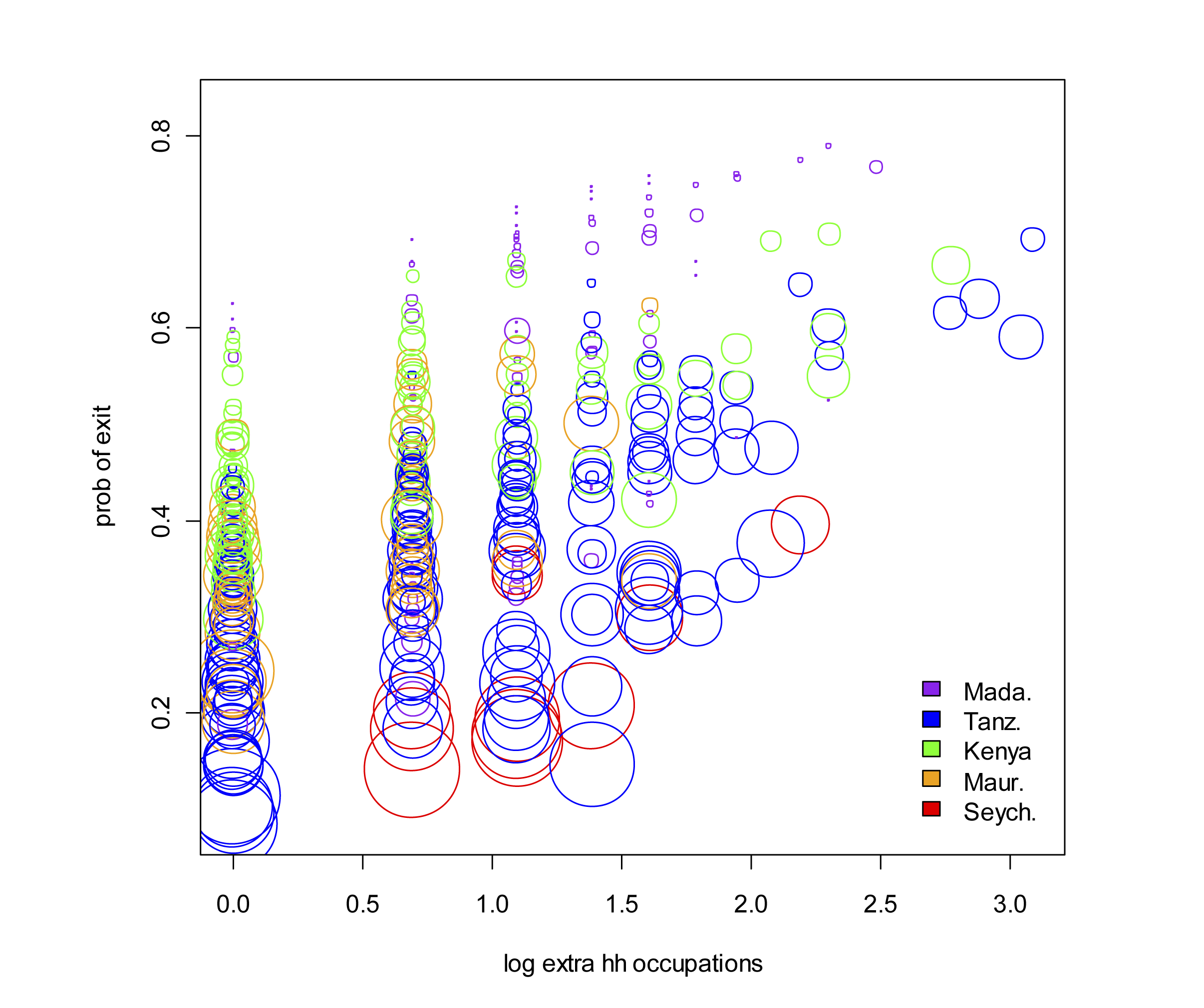


Figure S3. Relationships between the probability of a fisher stating they would exit from a hypothetical 50% decline in catch and the occupational multiplicity of their fellow householders, the value of their typical daily catch. Log-transformed normal day’s catch value is indicated by size of circles. Each circle shows the modelled probability of a fisher saying they would exit based on a generalised linear mixed-effects model. Scatter along the y-axis for given levels of predictor variables is caused by a random effect on community. Circles are coloured according to nation.
