# Supplementary material for: To Fish or Not to Fish: Factors at Multiple Scales Affecting Artisanal Fishers' Readiness to Exit a Declining Fishery
Source: PLoS One. 2012 Feb 10;7(2):e31460. doi: 10.1371/journal.pone.0031460 (PMC3277441; doi:10.1371/journal.pone.0031460)
Supplement: Table S1 — Factors considered in previous empirical studies of fisher mobility and whether they were found to be significant (if statistically tested). (DOCX) [file pone.0031460.s004.docx]

**Supporting Information**

Table S1. Factors considered in previous empirical studies of fisher mobility and whether they were found to be significant (if statistically tested). m: mentioned in discussion, ?: statistically tested, R: relationship identified, r: relationship found in subsets of the data, +/-: positive or negative relationship between factor and mobility.

|  | Variables and indicators | Number of papers mentioning | Number of refs testing | Number of references detecting significant effects | Pollnac et al. 2001 | Cinner et al. 2009 | Ikiara and Odink 2000 | Bailey (1982) | Smith et al. (2005) | Panayotou & Panayotou(1986) | Ward and Sutinen (1994) | Pita et al. (2010) | Pradham & Leung (2004) | Terkla et al. 1988 |
| --- | --- | --- | --- | --- | --- | --- | --- | --- | --- | --- | --- | --- | --- | --- |
| Individual | **Age** | 6 | 5 | 2 | ? | ? |  | ? |  | R - |  | R - |  |  |
|  | **Education** | 7 | 6 | 3 | r + | ? | ? | R + |  | R - |  | ? |  |  |
|  | Gender | 1 | 1 | 1 |  |  |  |  |  | R - |  |  |  |  |
|  | Marital status | 2 | 2 | 1 |  |  |  |  |  | R - |  | ? |  |  |
|  | Religion | 1 | 0 | 0 |  |  |  |  |  | M |  |  |  |  |
|  | **Wealth** | 1 | 1 | 1 |  | R - |  |  |  |  |  |  |  |  |
|  | Living standard trends | 1 | 1 | 1 | r - |  |  |  |  |  |  |  |  |  |
|  | Caste and cultural identity | 3 | 0 | 0 |  |  |  |  | M |  |  |  |  | M |
|  | community attachment | 1 | 0 | 0 |  |  |  |  |  |  |  | m |  |  |
|  | Local resident | 1 | 1 | 1 |  |  |  |  |  |  |  |  | R - |  |
| Relation to and perceptions of fishing | **family tradition or not (was the father a fisher)** | 2 | 1 | 0 |  |  | ? |  |  |  |  |  |  |  |
|  | Experience | 3 | 3 | 2 | ? |  | R - |  |  |  | R + |  |  |  |
|  | **reason entered and continuing fishing** | 1 | 1 | 0 |  |  |  |  |  |  |  | ? |  |  |
|  | Job satisfaction/lifestyle preferences | 5 | 1 | 1 |  |  |  |  | M | M |  | r - |  | M |
|  | Highliner illusions' expectation of catch/earnings | 2 | 0 | 0 |  |  |  |  | M |  |  |  |  | M |
| Livelihood | **Importance (and role) of fishing for livelihood** | 4 | 3 | 2 | r - | ? |  |  | M |  |  | r - |  |  |
|  | Degree of subsistence/commercial | 1 | 1 | 0 |  | ? |  |  |  |  |  |  |  |  |
|  | **Occupational diversity** | 1 | 1 | 0 |  | ? |  |  |  |  |  |  |  |  |
|  | **household occupational multiplicity** | 1 | 1 | 1 |  | R + |  |  |  |  |  |  |  |  |
|  | Access to alternative incomes or not | 2 | 2 | 1 |  |  | ? |  |  |  |  | r + |  |  |
|  | Experience/knowledge of other occupations | 4 | 1 | 1 |  |  |  |  | M | M |  | r + |  | M |
|  | Perception of earning in fishing | 1 | 1 | 1 |  |  |  |  |  |  |  | r - |  |  |
|  | Land ownership | 2 | 1 | 0 |  |  |  | ? |  | M |  |  |  |  |
| Fishing Characteristics | Skipper | 1 | 1 | 1 |  |  |  |  |  |  |  | r - |  |  |
|  | **Vessel owner** | 4 | 3 | 2 |  |  | ? | R - |  |  |  | M | R - |  |
|  | **Capital investment** | 5 | 2 | 0 |  | ? | ? |  | m |  |  | M |  | M |
|  | Generalist versus specialist vessel operations | 1 | 1 | 0 |  |  |  |  |  |  | ? |  |  |  |
|  | Vessel characteristics (length, tonnage, age) | 3 | 2 | 1 |  |  |  |  |  |  | R |  | ? |  |
|  | Earnings, Profitability and profit function | 3 | 3 | 3 |  |  |  |  |  | R - | R - |  | R - |  |
| Resource | **Resource abundance (or aggregated CPUE)** | 2 | 2 | 2 |  |  |  |  |  |  | R - |  | R - |  |
|  | **Catch rate** | 2 | 2 | 1 |  | ? | R - |  |  |  |  |  |  |  |
|  | **Perceived catch rate trend** | 1 | 1 | 1 | r - |  |  |  |  |  |  |  |  |  |
| Context | Fleet size (crowding externality) | 2 | 2 | 2 |  |  |  |  |  |  | R + |  | R - |  |
|  | **Location** | 3 | 3 | 3 | R |  |  |  |  | R |  | R |  |  |
|  | **Isolation of community** | 4 | 1 | 1 |  |  |  |  | m | R - |  |  |  | M |
|  | Welfare safety nets and perverse incentives | 2 | 0 | 0 |  |  |  |  | m |  |  |  |  | M |
|  | **Local non-farm economy** (including wages) | 6 | 1 | 1 |  |  | M | m | m | R + |  |  |  | M |
|  | Institutional environment | 2 | 0 | 0 |  |  |  |  | m |  |  |  | m |  |
|  | Prices in alternative fisheries | 1 | 1 | 0 |  |  |  |  |  |  | ? |  |  |  |
|  | Surplus labour caused by productivity gains | 2 | 0 | 0 |  |  |  |  | m |  |  |  |  | M |
