# Supplementary material for: To Fish or Not to Fish: Factors at Multiple Scales Affecting Artisanal Fishers' Readiness to Exit a Declining Fishery
Source: PLoS One. 2012 Feb 10;7(2):e31460. doi: 10.1371/journal.pone.0031460 (PMC3277441; doi:10.1371/journal.pone.0031460)
Supplement: Table S2 — Communities surveyed and summary statistics for each. * Only targeted fisher surveys (no random household surveys) conducted in Stone Town or Mazizini. (DOCX) [file pone.0031460.s005.docx]

**Supporting Information**

Table S2. Communities surveyed and summary statistics for each. * Only targeted fisher surveys (no random household surveys) conducted in Stone Town or Mazizini.

|  |  | | Data | n random household surveys | % respondents changed job in previous 5 years | % respondents changed and prefer new job in 5yrs | % households rank fishing as 1^st^ livelihood | % households with salaried occupations | Infrastructure index | Biomass density (kg/ha) of fish on adjacent reefs | n fisher interviews | % fishers would exit due to 50% decline |
| --- | --- | --- | --- | --- | --- | --- | --- | --- | --- | --- | --- | --- |
| Kenya | 1 | | Bamburi | 31 | 46.7% | 16.7% | 6.5% | 35.5% | 0.855 | 84 | 17 | 41% |
|  | 2 | | Kuruwitu | 32 | 51.6% | 19.4% | 15.6% | 12.5% | -0.230 | 106 | 4 | 50% |
|  | 3 | | Mayungu | 29 | 50.0% | 35.7% | 51.7% | 3.5% | -0.583 | 197 | 14 | 50% |
|  | 4 | | Mijikenda | 35 | 36.4% | 27.3% | 76.5% | 5.9% | 1.143 | 197 | 27 | 56% |
|  | 5 | | Shela | 31 | 33.3% | 16.7% | 25.8% | 12.9% | 1.832 | 197 | 10 | 40% |
|  | 6 | | Takaungu | 111 | 36.4% | 19.2% | 26.1% | 17.1% | -0.430 | 77 | 34 | 50% |
|  | 7 | | Utange | 40 | 47.4% | 23.7% | 0.0% | 7.7% | -0.836 | 38 | 20 | 65% |
|  | 8 | | Vipingo | 63 | 55.2% | 24.1% | 19.0% | 42.9% | 0.535 | 87 | 14 | 36% |
|  |  | | **KY Total** | **372** | **44.6%** | **22.8%** | **27.7%** | **17.2%** | **0.286** | **123** | **140** | **50%** |
| MD | 9 | | Ambodilaitry Area | 53 | 3.7% | 4.0% | 41.2% | 4.0% | -1.096 | 295 | 26 | 58% |
|  | 10 | | NW Madagascar | 70 | 9.0% | 6.0% | 42.9% | 1.4% | -1.019 | 273 | 36 | 25% |
|  | 11 | | Sahasoa | 44 | 19.4% | 6.5% | 7.0% | 6.8% | -0.523 | 538 | 15 | 87% |
|  | 12 | | Tampolo | 43 | 17.5% | 12.5% | 21.0% | 4.7% | -0.946 | 879 | 16 | 88% |
|  | 13 | | Tanjona | 54 | 0.0% | 0.0% | 75.0% | 3.9% | -1.066 | 472 | 38 | 74% |
|  |  | | **MD Total** | **264** | **9.9%** | **5.8%** | **37.4%** | **4.2%** | **-0.930** | **491** | **131** | **60%** |
| MS | 14 | | Blue Bay | 58 | 3.9% | 5.9% | 4.8% | 79.0% | 0.382 | 123 | 5 | 20% |
|  | 15 | | Le Morne | 40 | 23.1% | 17.9% | 15.0% | 30.0% | 1.063 | 103 | 13 | 46% |
|  | 16 | | Pointe aux Piments | 87 | 4.8% | 7.1% | 8.0% | 46.0% | 0.969 | 112 | 19 | 58% |
|  | 17 | | Pointe des Lascars | 65 | 7.8% | 7.8% | 18.5% | 53.9% | -0.080 | 85 | 18 | 22% |
|  | 18 | | St Martin | 61 | 14.0% | 14.0% | 5.1% | 20.3% | 0.396 | 78 | 13 | 31% |
|  |  | | **MS Total** | **311** | **10.7%** | **10.6%** | **10.3%** | **45.8%** | **0.546** | **100** | **68** | **38%** |
| SZ | 19 | | Anse Volbert | 21 | 91.7% | 50.0% | 9.5% | 52.4% | 1.537 | 336 | 5 | 20% |
|  | 20 | | Belombre | 89 | 43.5% | 36.2% | 3.4% | 63.6% | 1.528 | 302 | 13 | 23% |
|  | 21 | | Grand Anse | 92 | 50.0% | 36.7% | 0.0% | 58.7% | 1.356 | 269 | 3 | 00% |
|  | 22 | | Roche Caiman | 87 | 23.8% | 23.8% | 2.4% | 77.7% | 1.490 | 260 | 6 | 17% |
|  |  | | **SZ Total** | **289** | **52.2%** | **36.7%** | **3.8%** | **63.1%** | **1.478** | **292** | **27** | **19%** |
| TZ | 23 | | Buyu | 45 | 27.3% | 20.5% | 38.6% | 2.3% | -0.352 | 191 | 17 | 41% |
|  | 24 | | Dar Es Salaam | 59 | 35.6% | 16.9% | 54.2% | 6.8% | 0.893 | 151 | 42 | 29% |
|  | 25 | | Mazizini | No community level livelihoods data* | | | | | 1.320 | 191 | 41 | 41% |
|  | 26 | | Mtangata | 151 | 35.7% | 24.3% | 43.0% | 1.4% | -0.436 | 443 | 66 | 36% |
|  | 27 | | Nyamanzi | 54 | 22.9% | 12.5% | 42.9% | 12.2% | 0.084 | 191 | 27 | 56% |
|  | 28 | | Stone Town | No community level livelihoods data* | | | | | 1.682 | 191 | 40 | 18% |
|  |  | | **TZ Total** | **309** | **30.4%** | **18.5%** | **44.7%** | **5.7%** | **0.532** | **226** | **233** | **35%** |
|  | | **Totals all data** | | **1545** | **30.4%** | **18.7%** | **25.1%** | **25.5%** | **34%** | **231** | **599** | **44%** |
