# Supplementary material for: To Fish or Not to Fish: Factors at Multiple Scales Affecting Artisanal Fishers' Readiness to Exit a Declining Fishery
Source: PLoS One. 2012 Feb 10;7(2):e31460. doi: 10.1371/journal.pone.0031460 (PMC3277441; doi:10.1371/journal.pone.0031460)
Supplement: Table S3 — Variables included in analysis and data summarised by country. (DOCX) [file pone.0031460.s006.docx]

**Supporting Information**

Table S3. Variables included in analysis and data summarised by country

| **Data** | **KY** | **MD** | **MS** | **SZ** | **TZ** | **All countries** |
| --- | --- | --- | --- | --- | --- | --- |
| Sample size | 140 | 131 | 68 | 27 | 233 | 599 |
| Prop Better Job | 22.8% | 5.8% | 10.6% | 36.7% | 18.5% | 18.7% |
| Prop Fishing HHs | 27.7% | 37.4% | 10.3% | 3.8% | 44.7% | 25% |
| Infrastructure | 0.286 | -0.930 | 0.546 | 1.478 | 0.532 | 0.3381 |
| Gear |  |  |  |  |  |  |
| Seine or ring net | 30.0% | 0.0% | 0.0% | 3.7% | 9.9% | 11.0% |
| Gillnet | 35.7% | 43.5% | 2.9% | 0.0% | 21.9% | 26.7% |
| Handline | 16.4% | 36.6% | 41.2% | 70.4% | 33.5% | 32.7% |
| Spear | 7.1% | 6.9% | 5.9% | 3.7% | 10.3% | 8.0% |
| Trap | 5.7% | 2.3% | 26.5% | 14.8% | 21.0% | 13.7% |
| Other | 5.0% | 10.7% | 23.5% | 7.4% | 3.4% | 7.8% |
| Own boat or gear | 32.9% | 58.8% | 69.1% | 40.7% | 58.4% | 52.9% |
| Average of Age (± SD) | 37.6 ±15.2 | 36.4 ± 13.4 | 45.4 ± 12.6 | 40.2 ± 14.0 | 40.0 ± 12.7 | 39.3 ± 13.8 |
| Average of YrsEducation (± SD) | 4.85±3.8 | 3.23±2.6 | 5.69±3.7 | 9.16±5.3 | 5.70±3.7 | 5.11±3.8 |
| Average of NatMSL (± SD) | -0.250±0.8 | -0.097±0.9 | -0.249±0.7 | -0.109±0.8 | 0.086±0.9 | -0.078±0.9 |
| Average of JobDiversity (± SD) | 1.27±0.4 | 2.88±0.9 | 1.17±0.3 | 1.44±0.6 | 1.62±0.7 | 1.76±0.9 |
| FishingIsPrimary | 98.6% | 93.1% | 89.7% | 66.7% | 97.4% | 94.5% |
| Why started fishing |  |  |  |  |  |  |
| Choice | 9% | 8% | 11% | 5% | 7% | 9.3% |
| Necessity | 51% | 68% | 11% | 2% | 107% | 55.9% |
| Tradition | 17% | 4% | 23% | 9% | 42% | 22.2% |
| Reef Fish Biomass | 122.875 | 491.4 | 100.2 | 291.75 | 226.1667 | 230.9 |
| Average of NormCatchUSppp (± SD) | 26.4±38.0 | 2.2±2.9 | 86.0±221.2 | 1453.7±1633.9 | 131.7±476.7 | 112.1±474.3 |
| PerceiveDecline | 59.0% | 56.6% | 72.0% | 52.9% | 78.4% | 80.9% |
