# Supplementary material for: To Fish or Not to Fish: Factors at Multiple Scales Affecting Artisanal Fishers' Readiness to Exit a Declining Fishery
Source: PLoS One. 2012 Feb 10;7(2):e31460. doi: 10.1371/journal.pone.0031460 (PMC3277441; doi:10.1371/journal.pone.0031460)
Supplement: Table S4 — Results of GLMM regression with all individual or household variables. (DOCX) [file pone.0031460.s007.docx]

**Supporting Information**

Table S4. Results of GLMM regression with all individual or household variables.

|  | **coef** | **se(coef)** | **z** | **Pr(>\|z\|)** |
| --- | --- | --- | --- | --- |
| (Intercept) | 0.604405 | 0.794773 | 0.76047 | 0.447 |
| OwnBoatOrGear | -0.045678 | 0.22741 | -0.20086 | 0.841 |
| Age | -0.006639 | 0.007526 | -0.88212 | 0.378 |
| YrsEducation | 0.028248 | 0.028419 | 0.994 | 0.32 |
| locMSL | 0.129122 | 0.136067 | 0.94896 | 0.343 |
| log(JobDiversity) | 0.009012 | 0.25635 | 0.03516 | 0.972 |
| FishingHouse | -0.48223 | 0.525352 | -0.91792 | 0.359 |
| log(HseOccupExtr+1) | 0.361747 | 0.151893 | 2.38159 | 0.017200* |
| WhyFishCatchoice | 0.108291 | 0.447026 | 0.24225 | 0.809 |
| WhyFishCatneccesity | 0.17572 | 0.328679 | 0.53462 | 0.593 |
| WhyFishCattradition | 0.099139 | 0.373502 | 0.26543 | 0.791 |
| log(NormCatchUSppp) | -0.276299 | 0.077782 | -3.55221 | 0.000382*** |
| GearCatgillnet | 0.324631 | 0.394147 | 0.82363 | 0.41 |
| GearCathandline | -0.113403 | 0.395512 | -0.28673 | 0.774 |
| GearCatother | -0.190164 | 0.534152 | -0.35601 | 0.722 |
| GearCatspear | -0.120206 | 0.506246 | -0.23744 | 0.812 |
| GearCattrap | 0.04306 | 0.455618 | 0.09451 | 0.925 |
| PercDecline | 0.099732 | 0.302055 | 0.33018 | 0.741 |
